# Duration of Dual Antiplatelet Therapy After Implantation of Drug-Coated Balloon

**DOI:** 10.3389/fcvm.2021.762391

**Published:** 2021-12-01

**Authors:** Yuxuan Zhang, Xinyi Zhang, Qichao Dong, Delong Chen, Yi Xu, Jun Jiang

**Affiliations:** ^1^Department of Cardiology, Second Affiliated Hospital, School of Medicine, Zhejiang University, Hangzhou, China; ^2^Department of Cardiology, Ningbo First Hospital, Ningbo, China

**Keywords:** drug-coated balloon, dual antiplatelet therapy, in-stent restenosis, *de novo* coronary artery disease, percutaneous coronary intervention

## Abstract

The drug-coated balloon (DCB) is an emerging percutaneous coronary intervention (PCI) device with theoretical advantages and promising results. Recent clinical observations have demonstrated that DCB tends to have both good efficacy and a good safety profile in the treatment of in-stent restenosis (ISR) for both bare-metal and drug-eluting stents (DES), *de novo* coronary artery disease (CAD), and other situation, such as high bleeding risk, chronic total occlusion, and acute coronary syndrome (ACS). Dual antiplatelet therapy (DAPT) has become an essential medication in daily clinical practice, but the optimal duration of DAPT after the implantation of a DCB remains unknown. At the time of the first *in vivo* implantation of paclitaxel-DCB for the treatment of ISR in 2006, the protocol-defined DAPT duration was only 1 month. Subsequently, DAPT duration ranging from 1 to 12 months has been recommended by various trials. However, there have been no randomized controlled trials (RCTs) on the optimal duration of DAPT after DCB angioplasty. Current clinical guidelines normally recommend the duration of DAPT after DCB-only angioplasty based on data from RCTs on the optimal duration of DAPT after stenting. In this review, we summarized current clinical trials on DCB-only angioplasty for different types of CADs and their stipulated durations of DAPT, and compared their clinical results such as restenosis, target lesion revascularization (TLR) and stent thrombosis event. We hope this review can assist clinicians in making reasonable decisions about the duration of DAPT after DCB implantation.

## Introduction

Drug-coated balloon (DCB) technology is a combined therapy that involves a balloon and drug to treat coronary lesions, eliminating stent thrombosis, and reducing the rate of restenosis by leaving no metal behind (1). Since 2001, the DCB has been tested experimentally (1), and was later clinically validated in small-randomized controlled trials (RCTs) on coronary in-stent restenosis (ISR) (2) and peripheral stenosis (3). This technology has played an increasingly important role in the field of percutaneous coronary intervention (PCI), and a variety of products have been developed (Table 1). Drug-coated balloon technology has demonstrated safety and efficacy in the treatment of ISR and is recommended by guidelines as Class 1 indication for the treatment of ISR (5–8). Meanwhile, an increasing number of clinical studies using DCB have shown promising results for the treatment of both small and large vessel *de novo* coronary artery disease (CAD), bifurcation lesions, and other variable disease subsets.

**Table 1:** Major current drug-coated balloon available in the market (4).

**Name**	**Manufacturer**	**Type**	**Dosage**	**Coating method**	**Release characteristics**
SeQuent please	B. Braun Melsungen AG, Berlin, Germany	Paclitaxel	3 μg/mm^2^	Matrix coating: paclitaxel + hydrophilic spacer (iopromide)	Inflate for at least 40 s to allow enough drug to be released into the vessel wall (4.5% of the drug remains on the balloon)
DIOR-II	Eurocor GmbH, Bonn, Germany	Paclitaxel	3 μg/mm^2^	1:1 mixture of aleuritic and shellolic acid with paclitaxel (shellac® coating)	Drug delivery by simple diffusion, inflate 20–30 s at normal pressure
Elutax	Aachen Resonance GmbH, Aachen, Germany	Paclitaxel	2 μg/mm^2^	Two layers of paclitaxel (the first on the inflated balloon and the second as a crystal power), without any excipient	10% of the drug remains on the balloon after an inflation of 30–60 s
RESTORE DCB	Cardionovum, Bonn, Germany	Paclitaxel	3 μg/mm^2^	Shellac	A short-term balloon-to-vessel wall contact time of 45 s is enough
Pantera Lux	Biotronik, Bulach, Switzerland	Paclitaxel	3 μg/mm^2^	Paclitaxel + butyryl-trihexyl citrate	Minimum inflation time is 30 s to allow enough drug to be released into the vessel wall
Danubio	Minvasys, Gennevilliers, France	Paclitaxel	2.5 μg/mm^2^	Paclitaxel + butyryl-trihexyl citrate	Minimum inflation time is 30 s to allow enough drug to be released into the vessel wall
Protégé and Protégé NC	Blue Medical, Helmond, Netherlands	Paclitaxel	3 μg/mm^2^	Drug component encapsulated in wings using Wing Seal Technology	Load secured to achieve the therapeutic window within 30 s inflation time, also available with non-compliant balloon
MagicTouch	Concept Medical, Surat, India	Sirolimus	1.27 μg/mm^2^	Sirolimus is encapsulated in a phospholipid bi-layer as drug carrier and in Nanocarriers configuration	Inflate for at least 45 s if clinically tolerated
IN.PACT Falcon	Medtronic, Inc., Santa Rosa, California, USA	Paclitaxel	3 μg/mm^2^	Crystalline coating: paclitaxel + urea (FreePac®)	Inflate 30–60 s at normal pressure to allow enough drug release into the vessel wall (4.7% of the drug remains on the balloon)
Agent	Boston Scientific, Natick, MA, USA	Paclitaxel	2 μg/mm^2^	Balanced hydrophobic and hydrophilic properties of TransPax, Fewer particulates are lost distally during the procedure	Inflate for at least 30 s to allow enough drug to be released into the vessel wall
AngiosculptX	Spectranetics, Colorado Springs, Colorado, USA	Paclitaxel	3 μg/mm^2^	Nordihydroguaiaretic acid excipient to facilitate drug transfer to tissue	Inflate for at least 30 s, Improved dilatation in calcified or resistant lesion using a scoring balloon
Chocolate touch	QT Vascular	Paclitaxel	3 μg/mm^2^	Crystalline paclitaxel coating with hydrophilic excipient	The pillows and grooves of the inflated Chocolate Touch balloon result in 20% more drug-coated surface compared to conventional balloons of the same size
Essential	Ivascular	Paclitaxel	3 μg/mm^2^	Microcrystalline coating	Inflation process must last from 30 s to 1 min

Dual antiplatelet therapy (DAPT) has become an essential medication in daily clinical practice; it combines aspirin and a P2Y_12_-receptor inhibitor following PCI and is needed for the primary prevention of stent thrombosis and the secondary prevention of ischemic thrombotic event. With the “leave nothing behind” strategy, based on the shorter period of inflammatory response without a metallic scaffold, this strategy offers the theoretical advantage of virtually eliminating the threat of thrombosis over both the short and long term. Therefore, one possible benefit for many patients using DCB-only angioplasty is a short duration of DAPT, in some cases only 4 weeks, such as in patients with a high bleeding risk (9).

However, it must be pointed out that all previous studies have not adequately addressed questions about the optimal duration of DAPT after DCB implantation. The purpose of this review is to outline different DAPT strategies and trials with the use of DCB for ISR, *de novo* lesions, and other specific situations and to explore the appropriate DAPT duration to assist clinical practice.

## Current Guidance

As first recommended by the German Consensus Group (10), DAPT is necessary for 4 weeks if the DCB is used as a stand-alone procedure, and 6–12 months of DAPT is recommended in combination with bare metal stent (BMS). Then, they formulated more detailed recommendations regarding DAPT duration. In cases of the treatment of an ISR, the patient should receive aspirin 100 mg in the long-term and additional clopidogrel 75 mg for 4 weeks after PCI in BMS and at least 4 weeks or the duration defined by the drug-eluting stent (DES) implantation date. After treatment of small vessel *de novo* coronary lesions, aspirin 100 mg should be given long-term and clopidogrel 75 mg is recommended for 4 weeks after PCI with DCB alone and for 3 months after DCB with additional spot BMS. Dual antiplatelet therapy is recommended for 4 weeks if only DCB without stenting is used for the treatment of a bifurcation lesion and 6–12 months in case stents are used before or after the DCB procedure. To treat acute coronary syndromes (ACS), the recommended duration of DAPT is 12 months regardless of the use of a BMS, DES, or DCB (11). The Italian Position Group gave similar recommendations regarding a DAPT duration of at least 1 month in the case of DCB-only treatment and 3 months in cases of the implantation of a BMS (12).

However, the European Society of Cardiology Guidelines on DAPT gave more conservative recommendations. In patients with stable CAD treated with DCB, DAPT for 6 months should be considered. Dual antiplatelet therapy for 3 months should be considered if patients with stable CAD are considered a high bleeding risk. In patients with stable CAD in whom 3-months DAPT poses safety concerns, DAPT for 1 month may be considered. As in patients with ACS treated with coronary stent implantation, DAPT with a P2Y_12_ inhibitor on top of aspirin is recommended for 12 months unless contraindicated. In cases of patients who are at high risk of bleeding, discontinuation of P2Y_12_ inhibitor therapy after 6 months should be considered (13).

Recently, the Asia-Pacific Consensus Group updated the DCB treatment protocols for CAD and gave their recommendations regarding optimal medical treatment. For the treatment of BMS-ISR and DES-ISR, patients should maintain a lifelong therapy with aspirin 100 mg and take clopidogrel 75 mg for at least 1–3 months. For the treatment of *de novo* coronary disease except ACS with DCB only, patients should receive DAPT for at least 1 month and then receive aspirin 100 mg for life. Moreover, in cases of *de novo* stable coronary disease with DCB plus bail-out BMS, DAPT is recommended for at least 3–6 months. For the treatment of bifurcation disease, if the DCB-only method without stenting is used, the duration of DAPT should be the same as other *de novo* coronary disease. In the case of the DCB method plus stenting, the recommended DAPT duration is at least 6–12 months. For patients with ACS, similar to other guidelines, DAPT is recommended for at least 12 months regardless of the use of BMS, DCB, or DES (7).

## Pharmacology of Antiplatelets

**Figure 1:**
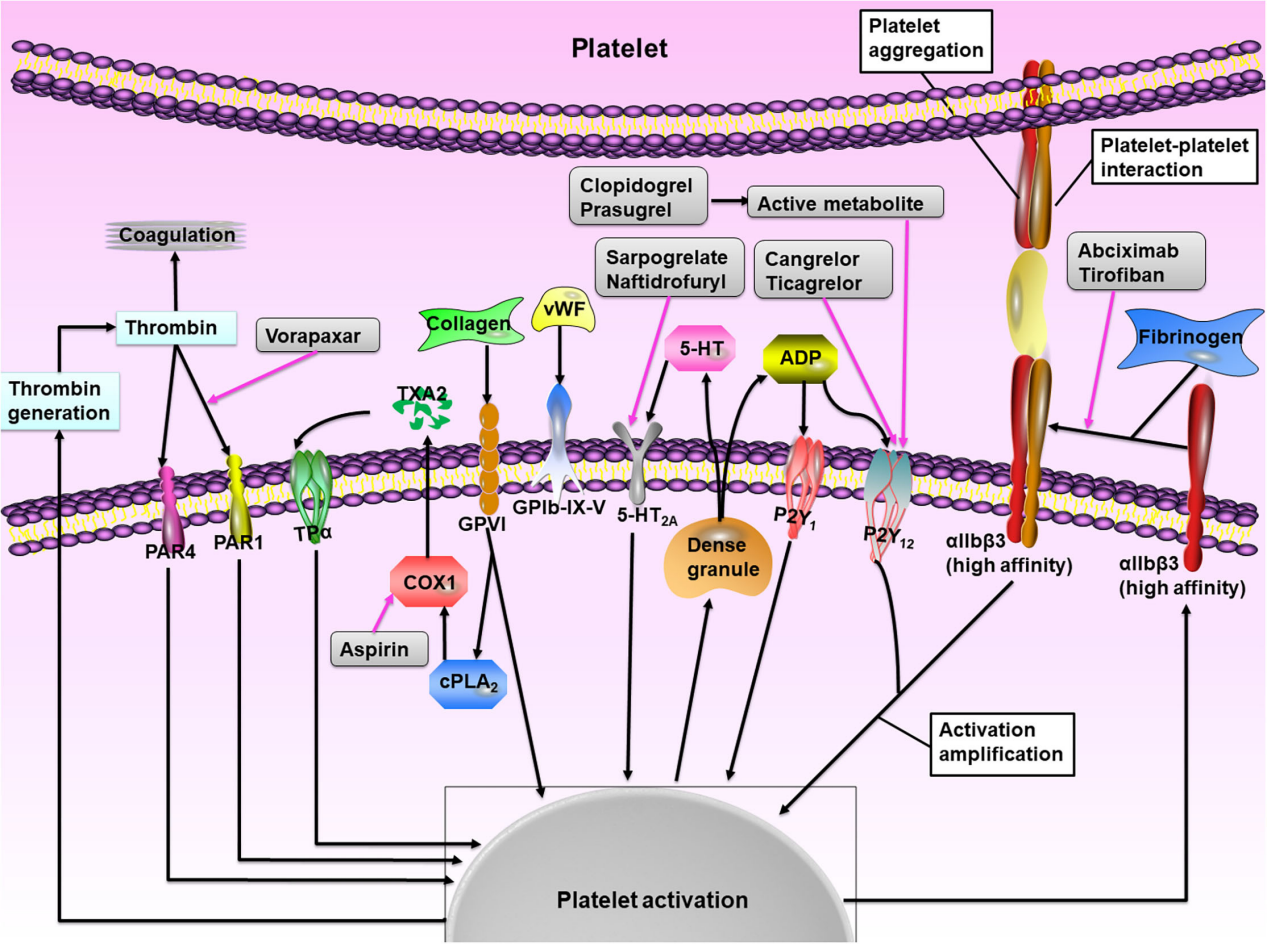
The role of platelet activation. At the site of vascular injury, platelet adherence to the endothelium through the combination of glycoprotein (GP) receptors with exposed extracellular matrix proteins (particularly collagen and von Willebrand factor, vWF). Platelet activation occurs through complex intracellular signaling processes and leads to the release of various agonists, including thromboxane A_2_ (TXA_2_), ADP, and 5-hydroxytryptamine (5-HT), which act by binding to their respective G protein-coupled receptors and mediate paracrine and autocrine platelet activation. The receptor P2Y purinoceptor 12 (P2Y_12_) has a major role in the amplification of platelet activation, which is also supported by outside-in signaling via αIIbβ3 integrin (the glycoprotein IIb/IIIa receptor). The main platelet integrin GPIIb/IIIa mediates platelet aggregation through conformational shape changes and binding to fibrinogen and vWF, thereby mediating the final common step of platelet activation. The net result of these interactions is thrombus formation mediated by the interaction of platelet aggregate with fibrin and thrombin. The available drugs (gray boxes) blockade different pathways of platelet activation and show additive inhibitory effects when used in combination. PAR, proteinase-activated receptor; TPα, TXA_2_ receptor isoform-α; COX1, cyclooxygenase 1. Adapted from Varga-Szabo et al. (14).

The goal of antiplatelet therapy after PCI is to maximize protection against short- and long-term postoperative stent or vessel thrombosis by blocking platelet activation while limiting bleeding risk. Figure 1 illustrates the main mechanisms of platelet activation and the sites of action of antiplatelet agents. Platelet adhesion is mediated by the interaction between platelet receptors and ligands exposed at the sites of vascular injury, e.g., the glycoprotein (GP) Ib/V/IX receptor complex with the von Willebrand factor and GPVI and GPla proteins with collagen (15–17). Then the local platelet activating factors, such as adenosine diphosphate (ADP), thromboxane A_2_ (TXA_2_), serotonin, and thrombin, promote and amplify the platelet activation by interacting with specific platelet membrane receptors [such as P2Y purinoceptor 12 (P2Y_12_), 5-hydroxytryptamine 2A receptor, TXA_2_ receptor isoform-α, and proteinase-activated receptors (PARs)] (15–17). Antiplatelet drugs block platelet activation through different phases: (1) Acetylsalicylic acid, commonly known as aspirin, is an irreversible cyclooxygenase 1 (COX1) inhibitor that diminishes platelet activation and aggregation promoted by TXA_2_ by blocking TXA_2_ production during pathological thrombus formation: (2) P2Y_12_ ADP receptor antagonists, which include clopidogrel, prasugrel, and ticagrelor, exert their clinical benefit by inhibiting the activation of P2Y_12_-mediated platelet activation during pathological thrombosis (when the occlusive platelet-rich thrombus is formed); (3) Glycoprotein IIb/IIIa inhibitors, including eptifibatide and tirofiban, are currently only for ACS patients undergoing PCI, and interfere with platelet cross-linking and clot formation by competing with fibrinogen and vWF for GP IIb/IIIa binding; (4) Vorapaxar, as a PAR-1 inhibitor, blocks the binding of thrombin to PAR-1, thus inhibiting thrombin-induced activation, and the aggregation of platelets; (5) Cilostazol is an inhibitor of phosphodiesterase type III, which possesses both antiplatelet and vasodilatory effects (15–17).

## DAPT Duration in Multiple Diseases

### In-stent Restenosis

In-stent restenosis remains the primary cause of PCI failure, though the development of DESs generations has improved anti-restenosis performance (18). A recent report showed that approximately 20% of patients required target lesion revascularization (TLR) at the 10-year follow-up (19). Several therapies for ISR of BMS or DES have been tested in clinical trials (20), and DCB and repeated stenting with DES have become the most effective therapeutic options, which have been recommended as class IA by guidance (5). A recent meta-analysis of 10 RCTs showed that DCB and DES were similarly effective and safe in the treatment of BMS-ISR, whereas DES had higher efficacy than DCB in the treatment of DES-ISR (21).

As the first trial demonstrated that paclitaxel-DCB angioplasty was superior to plain old balloon angioplasty (POBA) alone in BMS-ISR, the PACCOCATH ISR trial recommended DAPT for 1 month followed by treatment with aspirin alone (2). At 6 months, the primary endpoint late lumen loss (LLL) in-segment was lower in the DCB group than in the POBA group (0.03 ± 0.48 mm vs. 0.74 ± 0.86 mm, *P* = 0.002). Restenosis occurred in 10 of 23 patients (43%) in the POBA group, compared to only 1 of 22 patients (5%) in the DCB group (*P* = 0.002). Patents who required TLR were significantly fewer in the DCB group than in the POBA group (0 vs. 6%, *P* = 0.02). At 5 years, TLR rates were still significantly lower in the DCB group than in the POBA group (38.9 vs. 9.3%, *P* = 0.004) (22). No stent thrombosis was found during the entire follower-up trial, which suggests that short-term DAPT may be feasible and safe for patients who undergo DCB angioplasty.

In most trials on BMS-ISR treatment, DAPT with aspirin 100 mg per day and clopidogrel was recommended for 3 months (23–26) (Table 2). Among these trials, only two cases of stent thrombosis were found during the follow-up (24, 25). It is worth mentioning that one patient in the DCB group experienced stent thrombosis due to clopidogrel discontinuation before late angiography in the RIBS V trial. The PEPCAD II ISR study showed that 19 of 66 (28.8%) patients in the DCB group and 42 of 65 (64.4%) patients in the DES group were still using clopidogrel (*P* < 0.0001) at 6 months, whereas after 12 months, the usage declined to 12 of 66 (18.1%) and 27 of 65 (41.5%), respective (*P* < 0.01) (23). However, there was no significance difference between the treatment groups with DAPT at 1 and 3 years (*P* = 0.80 and 0.47, respectively) (35).

**Table 2:** Characteristics of randomized trials of DCB for treatment of ISR.

**Trial (year)**	**Patients, N**	**Design**	**DAPT duration (months)**	**Primary endpoint (follow-up, months)**	**Binary restenosis rate, %**	**TLR, % (follow-up, months)**	**ST, *N* (follow-up, months)**
***BMS-ISR***							
PACCOCATH ISR (2006) (2)	52	DCB vs. POBA	1 in both groups	LLL: 0.03 ± 0.48 mm vs. 0.74 ± 0.86 mm^*^ (6)	5 vs. 43%^*^	0 vs. 23%^**^ (12)	0 vs. 0 (12)
PEPCAD II isr (2009) (23)	131	DCB vs. PES	3 in DCB vs. 6 in PES	LLL: 0.17 ± 0.42 mm vs. 0.38 ± 0.61 mm (6)	7 vs. 20%	6.3 vs. 15.4% (36)	0 vs. 0 (36)
ribs V (2014) (24)	189	DCB vs. EES	3 in DCB vs. 12 in EES	MLD: 2.01 ± 0.6 mm vs. 2.36 ± 0.6 mm^*^ (9)	9.5 vs. 4.7%	6 vs. 1% (12) 8 vs. 2%^**^ (36)	1 vs. 0 (36)
PATENE-C (2016) (25)	61	PCSB vs. USB	3 in both	LLL: 0.17 ± 0.40 mm vs. 0.48 ± 0.51 mm^*^ (6)	7 vs. 41%^*^	3 vs. 32%^*^ (12)	0 vs. 0 (12)
Pleva et al. (2016) (26)	136	DCB vs. EES	3 in DCB vs. 6-12 in EES	LLL: 0.09 ± 0.73 mm vs. 0.44 ± 0.73 mm^*^ (12)	8.7 vs. 19.12%	7.35 vs. 16.18% (12)	1 VS. 0 (12)
***DES-ISR***							
PEPCAD-DES (2012) (27)	110	DCB vs. POBA	6 in both groups	LLL: 0.43 ± 0.61 mm vs. 1.03 ± 0.77 mm^*^ (6)	17.2 vs. 58.1%^*^	15.3 vs. 36.6%^*^ (6) 19.4 vs. 36.8%^**^ (36)	1 vs. 4^**^ (36)
ISAR-DESIRE 3(2013) (28)	402	DCB vs. PES vs. POBA	6 in all groups	DS: 38.0% in DCB vs. 37.4% in PES vs. 54.1% in POBA (6–8)	NA	22.1% in DCB vs. 13.5% in PES vs. 43.5% in POBA (12) 33.3% in DCB vs. 24.2% in PES vs. 50.8% in POBA (36)	1 vs. 1 vs. 0 (12) 1 vs. 2 vs. 0 (36)
Pepcad China ISR (2014) (29)	220	DCB vs. PES	12 in both groups	LLL: 0.46 ± 0.51 mm vs. 0.55 ± 0.61 mm (9)	18.6 vs. 23.8%	15.6 vs. 12.3% (12) 15.9 vs. 13.7% (24)	1 vs. 2 (12) 1 vs. 3 (24)
Ribs IV (2015) (30)	309	DCB vs. EES	3 in DCB vs. 12 in EES	MLD: 1.80 ± 0.6 mm vs. 2.03 ± 0.7 mm^*^ (6–9)	19 vs. 11%	13.0 vs. 4.5%^*^ (12) 15.6 vs. 7.1%^**^ (36)	3 vs. 2 (12) 4 vs. 2 (36)
ISAR-DESIRE 4 (2017) (31)	252	DCB vs. SB-DCB	6 in both groups	DS: 40.4 ± 21.4 vs. 35 ± 16.8%^**^ (6–8)	32.0 vs. 18.5%^**^	21.8 vs. 16.2% (12)	0 vs. 0 (12)
Restore (2018) (32)	172	DCB vs. EES	6 in both groups	LLL: 0.15 ± 0.49 mm vs. 0.19 ± 0.41 mm (9)	19.5 vs. 5.6%	5.8 vs. 1.2% (12)	0 vs. 0 (12)
***Both BMS-ISR and DES-ISR***							
DARE (2018) (33)	278	DCB vs. EES	12 in both groups	MLD: 1.71 ± 0.51 vs. 1.74 ± 0.61 (6)	18.1 vs. 20.9%	8.8 vs. 7.1% (12)	0 vs. 0 (12)
Blolux (2018) (34)	229	DCB vs. SES	Given as per local standard	LLL: 0.03 ± 0.40 mm vs. 0.20 ± 0.70 (6)	NA	13.5 vs. 11.6% (18)	1 vs. 2 (18)

*DAPT, dual anti-platelet therapy; TLR, target lesion revascularization; ST, stent thrombosis including definite and possible; DCB, drug-coated balloon; POBA, plain old balloon angioplasty; LLL, late lumen loss; PES, paclitaxel-eluting stent; EES, everolimus-eluting stent; MLD, minimal lumen diameter; PCSB, paclitaxel-coated scoring balloon; USB, uncoated scoring balloon; DS, diameter restenosis; SB-DCB, scoring balloon before drug-coated balloon; SES, sirolimus-eluting stent*.

* *P < 0.01 vs. non-DCB group*.

** *P < 0.05 vs. non-DCB group*.

However, the duration of DAPT varied from 3 to 12 months in trials for DES-ISR treatment (Table 2). In the RIBS IV randomized clinical trial, which showed DCB had lower efficacy compared to EES in patients presenting with DES-ISR, DAPT was prescribed for only 3 months after DCB angioplasty, and then aspirin monotherapy was maintained (30, 36). The TLR rates were significantly reduced in the EES group both at 1-year (4.5 vs. 13.0%, *P* = 0.007) and 3-years (7.1 vs. 15.6%, *P* = 0.015) follow-up, but the need for “late” (>1 year) TLR (2.6 vs. 4%) was similar in the two groups. Stent thrombosis (both definitive and probable) occurred in three patients (two in the DCB group and one in the EES group) at 1 year and after that another two cases of stent thrombosis occurred in the DCB group at 3 years (36). However, during the actual follow-up for the trial, 84% of patients in the DCB group were still receiving DAPT at 9 months, and 64% were still receiving DAPT at 1 year, of which 52% suffered from unstable angina at the time of recruitment. For most trials, DAPT was administered for 6 months after DCB dilatation (27, 28, 31, 32). In these trials, DCB showed higher efficacy than POBA with DES-ISR treatment, which showed similar efficacy to DES. Two stent thrombosis cases were found in the DCB group during the follow-up, one in the PEPCAD-DES trial (37) and another in the ISAR-DESIRE trial (28). The PEPCAD China ISR trial also demonstrated that angioplasty with DCB was non-inferior to PES implantation when used to treat DES-ISR (29, 38). In this trial, all patients, irrespective of treatment allocation, were prescribed DAPT for 12 months. There was one late stent thrombosis occurred in the DCB group and two in the PES group at the 1-year follow-up, and another very late stent thrombosis occurred in the PES group at the 2-year follow-up (38).

### Small Vessel *de novo* Coronary Artery Disease

It remains challenging to treat coronary small-vessel disease, which is usually defined as lesions in vessels <3.0 or ≤ 2.75 mm, because it is significantly and directly associated with an increased risk of clinical events (39). Though DES has been found to be equally effective in small and large vessels, the resulting LLL occupies a higher percentage of the respective vessel diameter, leading to a higher incidence of ISR and other clinical events (40). Drug-coated balloon angioplasty has the theoretical advantage of providing immediate and homogenous drug uptake, leaving no metal in the coronary artery and respecting the vessel anatomy, thus forming a “leave nothing behind” strategy in the treatment of *do novo* CAD (41). Many notable RCTs involving small vessel disease have used this strategy and all studies have shown the benefits of DCB except the PICCOLETO (42) (Table 3), which may be explained as the limitations of the first-generation Dior DCB (49). A recent meta-analysis showed that the use of DCB in the treatment of *do novo* CAD was associated with comparable clinical outcomes regardless of the indication or comparator device (50). However, there is still no clear conclusion regarding the duration of DAPT against small-vessel disease treated by DCB.

**Table 3:** Characteristics of randomized control trials of DCB for treatment of small vessel *de novo* coronary artery disease.

**Trial (year)**	**Patients, *N***	**Design**	**DAPT duration (months)**	**Primary endpoint (follow-up, months)**	**Binary restenosis rate, %**	**TLR, % (follow-up, months)**	**ST, N (follow-up, months)**
PICCOLETO (2010) (42)	57	DCB vs. DES	1 in SAP and alone DCB use vs. 3 in DCB + stent implantation vs. 12 in UAP or DES	DS: 43.6 vs. 24.3%^**^ (6)	32.1 vs. 10.3%^**^	32.1 vs. 10.3% (9)	0 vs. 0 (9)
Bello (2012) (43)	182	DCB vs. PES	1 in DCB only vs. 3 in DCB + BMS vs. 12 in PES	LLL: 0.08 ± 0.38 mm vs. 0.29 ± 0.44 mm^*^ (6)	8.9 vs. 14.1%	4.4 vs. 7.6% (6) 6.7 vs. 13% (36)	0 vs. 0 (36)
Funatsu et al. (2017) (44)	135	DCB vs. POBA	3 in both groups	TVF: 3.4 vs. 10.3% (6)	13.3 vs. 42.5%^*^	2.3 vs. 10.3% (6)	0 vs. 0 (6)
BASKET-SMALL 2 (2018) (45)	758	DCB vs. nDES	1 in SAP and DCB only vs. 6 in SAP and DES vs. 12 in ACS vs. 3 in DCB + BMS vs. 6 in DCB + DES	MACE: 7.3 vs. 7.5% (12) MACE: 15 vs. 15% (36)	NA	3.4 vs. 4.5% (12) 9 vs. 9% (36)	2 vs. 4 (13) 2 vs. 6 (36)
Angiographic analysis from the BASKET-SMALL 2 (2020) (46)	111	ditto	ditto	DS: 35.8 vs. 29.0%^**^ (median 5.7)	20.4 vs. 21.5%	NA	NA
Restore SVD China (2018) (47)	230	DCB vs. nDES	At least 6 in both groups	DS: 29.6 ± 2.0 vs. 24.1 ± 2.0% (9)	11.0 vs. 8.6%	4.4 vs. 2.6% (12) 5.2 vs. 2.8% (24)	0 vs. 0 (24)
PICCOLETO II (2020) (48)	232	DCB vs. EES	1 in SAP and DCB vs. 6 in EES vs. 12 in ACS	LLL: 0.04 ± 0.28 mm vs. 0.17 ± 0.39 mm^**^ (6)	6.3 vs. 6.5%	5.6 vs. 5.6% (12)	0 vs. 2 (12)

*DAPT, dual anti-platelet therapy; TLR, target lesion revascularization; ST, stent thrombosis including definite and possible; DCB, drug-coated balloon; DES, drug-eluting stent; SAP, stable angina pectoris; UAP, unstable angina pectoris; DS, diameter restenosis; PES, paclitaxel-eluting stent; BMS, bare-metal stent; POBA, plain old balloon angioplasty; TVF, target vessel failure; nDES, new-generation drug-eluting stent; ACS, acute coronary syndrome; MACE, major adverse cardiac events; EES, everolimus-eluting stent*.

* *P < 0.01 vs. non-DCB group*.

** *P < 0.05 vs. non-DCB group*.

At present, the widely used postoperative DAPT strategy from clinical trials for the DCB treatment of small-vessel disease is the following: (1) DAPT duration in stable patients using DCB is 4 weeks; (2) DAPT duration in stable patients using DES is 6 months; (3) DAPT duration in patients with ACS is 12 months; and (4) DAPT duration in patients treated with a combination of DCB and BMS is 3 months, and in patients with DCB and DES is 6 months. Notable trials that used this strategy include PICCOLETO (42), BELLO (43), BASKET-SMALL 2 (45), and PICCOLETO II (48). Among these trials, the binary restenosis rates and TLR rates were comparable between the DCB and DES groups and were all low-probability events. It is important to acknowledge that patients treated with DCB and without stenting did not experience any thrombotic events in these trials, whereas only two stent thrombosis events were found in the BASKET-SMALL 2 trial during the 3-year follow-up (45). These results suggest that DCB may provide significant advantages over DES in treating small vessel disease, such as a lower risk of stent thrombosis, a shorter duration and less dependence on DAPT (43).

The RESTORE SVD China trial also demonstrated that the Restore DCB was non-inferior to the second-generation RESOLUTE Integrity DES (47, 51). However, DAPT was prescribed for at least 6 months after discharge from the hospital. During the 12-month follow-up, no significant difference was observed in the comparison of DAPT duration between the DCB and DES groups (91.4 vs. 94.7%), which was partly due to the high proportion of unstable angina in this study and the high incidence of MACE in small vessels.

### Large Vessel *de novo* Coronary Artery Disease

Many interventional cardiologists had doubts about the safety of DCB alone for large vessel *de novo* CAD because large coronary arteries have more smooth muscle fibers than small vessel arteries and are more prone to recoil and dissection, which may lead to acute occlusion or restenosis of blood vessels (52). Although randomized data for comparing DCB and DES in the treatment of large vessels are still lacking, there are variable proportions of large vessels that were treated using the DCB-only approach in studies, which creates growing evidence for the safety and efficacy of the DCB-only strategy for the treatment of large coronary arteries. The durations of DAPT in these trials ranged from 1 to 12 months (Table 4).

**Table 4:** Characteristics of prospective trials of DCB for treatment of *de novo* coronary artery disease including large vessels.

**Trial (year)**	**Patients, *N***	**Design**	**DAPT duration (months)**	**RVD, mm**	**Primary endpoint (follow-up, months)**	**TLR, % (follow-up, months)**	**ST, N (follow-up, months)**
**Randomized control trial**
Nishiyama et al. (2016) (53)	60	DCB vs. EES	8 in both groups	2.88 ± 0.57 mm vs. 2.72 ± 0.64 mm	LLL: 0.25 ± 0.25 mm vs. 0.37 ± 0.40 mm (8)	0.0 vs. 6.1% (8)	NA
Gobić et al. (2017) (54)	75	DCB vs. SES	12 in both groups	2.61 ± 0.49 mm vs. 3.04 ± 0.46 mm	LLL: −0.09 ± 0.08 mm vs. 0.10 ± 0.19 mm^**^ (6)	0.0 vs. 5.4% (6)	0 vs. 2 (6)
REVELATION (2019) (55)	120	DCB vs. DES	9 in both groups	3.28 ± 0.52 mm vs. 3.20 ± 0.48 mm	FFR: 0.92 ± 0.05 vs. 0.91 ± 0.06 (9)	3 vs. 2% (9)	1 vs. 0 (9)
DEBUT (2019) (9)	220	DCB vs. BMS	1 in both groups	NA	MACE: 1 vs. 14%^*^ (9) MACE: 4 vs. 14%^**^ (12)	0 vs. 6%^*^ (9) 2 vs. 6% (12)	0 vs. 2 (12)
**Prospective study**
Cortese et al. (2015) (56)	156	DCB	1 in DCB only vs. 6 in DCB and stent implantation	2.83 (2.12–3.01) mm	Complete vessel healing rate: 93.8% (6)	6.2% in dissection cohort vs. 5.3% in ALL DCB	NA
Shin et al. (2016) (57)	66	DCB vs. nDES	1.5 in DCB vs. 12 in DES vs. 6 in BMS	2.69 ± 0.45 mm vs. 2.92 ± 0.31 mm	LLL: 0.05 ± 0.27 mm vs. 0.40 ± 0.54 mm^**^ (9)	0.0 vs. 4.5% (12)	0 vs. 0 (12)
Ann et al. (2016) (58)	27	DCB	1.5	2.58 ± 0.45 mm	LLL: 0.02 ± 0.27 mm (9)	0.0% (9)	NA
Lu et al. (2019) (59)	92	DCB	6	3.32 ± 0.46	LLL: −0.02 ± 0.49 mm (9)	4.3% (12)	NA
Rosenberg et al. (2019) (60)	686	DCB	1 in DCB vs. 6 in DCB + stent implantation	2.31 ± 0.26 mm in small vessels vs. 3.16 ± 0.26 mm in large vessels	TLR: 2.4% in small vessels vs. 1.8% in large vessels (9)	TLR: 2.4% in small vessels vs. 1.8% in large vessels (9)	1 in small vessels vs. 1 in large vessels (9)

*DAPT, dual anti-platelet therapy; RVD, reference vessel diameter; TLR, target lesion revascularization; ST, stent thrombosis including definite and possible; DCB, drug-coated balloon; EES, everolimus-eluting stent; LLL, late lumen loss; DES, drug-eluting stent; FFR, fractional flow reserve; SES, sirolimus-eluting stent; BMS, bare-metal stent; MACE, major adverse cardiac events*.

* *P < 0.01 vs. non-DCB group*.

** *P < 0.05 vs. non-DCB group*.

A recent prospective large-scale multicenter trial demonstrated that DCB as a stand-alone-therapy showed similar efficacy on large and small vessels (60, 61). In this trial, standard DAPT duration was recommended for 1 month in DCB-only treatment and a minimum of 6 months when additional stents were implanted. During the follow-up, in the large vessel group, the DAPT duration was 2.7 ± 1.6 months, whereas in the small vessel group, the DAPT duration was 2.8 ± 1.6 months (*p* = 0.583). Meanwhile, this trial observed that around half of each group had a recommendation for 4 weeks of DAPT (>2.75 mm: 53.3% vs. ≤ 2.75 mm: 48.1%, *p* = ns). Only one case of stent thrombosis occurred in each group. The DEBUT trial which showed DCB-only coronary intervention was superior to BMS in patients at bleeding risk, was also administered 1-month of DAPT for all patients (9). In patients assigned to DCB, 64% were treated with a DCB that was 3 mm or larger diameter, and stent thrombosis occurred in none of them. A short duration of DAPT after DCB angioplasty was recommended in the other three trials without any stent thrombosis found and low risk rates of clinical events (56–58). These results indicate that short-term DAPT may be feasible and safe. On the contrary, some trials recommended DAPT for 6 months or longer, and they also did not find stent thrombosis during follow-up (53–55). These trials chose a longer DAPT duration because most patients in these trials were admitted to the hospital for ACS.

### Other Clinical Situations

#### Chronic Total Occlusions

Chronic Total Occlusions (CTOs) of the coronary arteries remain one of interventional cardiologists' biggest challenges and some scholars have also attempted to apply the DCB-only strategy to CTO (62, 63). A prospective trial led by Köln et al. showed that the DCB-only strategy as a treatment option for CTO was feasible and well-tolerated (63). Most patients received DAPT for at least 4 weeks and 4 of 34 patients had contraindications for DAPT who received only lifelong aspirin in this trial. Restenosis occurred in 11.8% of all patients, re-occlusion in 5.9%, TLR in 17.6%, and no stent thrombosis was found.

#### High Bleeding Risk

An all-comers retrospective study that contained 52% high-bleeding-risk patients showed the safety and feasibility of short-term DAPT after DCB angioplasty for both stable CAD and ACS (64). The median and mean durations of DAPT were 1 and 2.8 months in the stable CAD population and 1 and 3.3 months in the ACS population. The MACE rate was 9.8 and 14.8% at 12 and 24 months with 2.1 and 3.1% TLR rates, respectively. Recently, the DEBUT trial demonstrated that DCB was superior to BMS for the treatment of *de novo* coronary artery lesions in patients with high bleeding risk (9). The duration of DAPT was 1 month in patients with stable CAD and ACS in both groups. For ACS patients receiving anticoagulation therapy, the duration of aspirin was 6 months. At 9 months, the MACE and TLR rates were 1 and 0% in the DCB group and 14 and 6% in the BMS group, respectively. One case of stent thrombosis occurred in each group.

### Duration in ACS

Although receiving second-generation DES is the most common option for the treatment of patients with ACS and is generally considered the optimal strategy (65), some small sample size clinical trials have attempted to use the DCB-only strategy in primary percutaneous coronary intervention (PPCI) (54, 55, 66–68). Nicola et al. conducted the first study of a DCB-only strategy in the setting of PPCI, and DPAT was scheduled to be continued for 12 months (66). This study showed good 1-year clinical results with only five MACEs occurring, including three TLR and one acute stent thrombosis, but additional stenting was performed in half of the patients. Recently, the REVELATION study shown that DCB was non-inferior to the second-generation DES in the treatment of ST-segment elevated myocardial infarction patients (55). All patients were on DAPT and/or combined with oral anticoagulation for at least 1 year. Up to the 9-month follow-up, only three patients required TLR (one in the DES group and two in the DCB group) and only one thrombotic event was found in the DCB group. The 1-year duration of DAPT seemed to be a reasonable option based on the guideline recommendations and the results of existing clinical trials.

However, it remains a question whether DAPT duration for ACS patients is worth reducing or prolonging. A recent meta-analysis of the duration of DAPT after PCI with DES demonstrated that short-term DAPT presented similar efficacy and safety to standard-term DAPT for patients with ACS (69). In the DEBUT trial, 46% of patients treated with DCB only were diagnosed with ACS (9). All patients in this trial were recommended to undergo only a 1-month duration of DAPT, and the results showed low MACE rates with no TLR event at 9 months. Meanwhile, no stent thrombosis event occurred during the follow-up. In another retrospective study which contained 55% of ACS patients showed a 12% MACE rate and 2.8% TLR rate at 12 months (64). The median and mean durations of DAPT were 1 and 3.3 months in the ACS population. Of note, half of the patients had at least one risk factor for bleeding. Furthermore, about 4% of patients did not receive any ADP receptor blockers at all during or after PCI due to a contraindication for DAPT. Meanwhile, the European Society of Cardiology Guidelines also suggest that patients at high or moderate ischemic risk who have well-tolerated DAPT within the first year after myocardial infarction may benefit from more intense antithrombotic therapy beyond 12 months from the acute event (70). For this kind of patients (e.g., age ≥65 years and multivessel coronary disease), aspirin 75–100 mg with ticagrelor 60 mg twice daily or rivaroxaban 2.5 mg twice daily may be administered, which would reduce the ischemic risk with no major bleeding events and infrequent minor/minimal bleeding (71, 72). Whether prolonging or reducing the duration of DAPT, it is important to tailor the treatment to each patient to get the best benefits of DAPT.

### Duration for New-Generation Sirolimus DCB

Thus far, paclitaxel, as a cytotoxic agent, is the preferred drug for balloon coating and has been widely cited in cardiovascular interventional therapies. With the growing clinical research evidence of sirolimus-coated balloon (SCB), their clinical feasibility and safety are being increasingly recognized. The SABRE trial showed excellent procedural success for the Virtue sirolimus-eluting angioplasty balloon in the treatment of ISR (73). In this trial, DAPT was continued for at least 3 months and the MACE rate was 14.3% at 12 months, with a 12.2% TLR rate. Soon after that, an RCT that compared a crystalline coating SCB with paclitaxel-coated balloon (PCB) demonstrated similar angiographic outcomes in the treatment of coronary DES-ISR (74). Interestingly, DAPT was recommended for 1 months in stable patients and 12 months in cases of ACS. The MACE and TLR rates were similar between the two groups, and only one stent thrombosis occurred in the PCB group. Two other large prospective trials that enrolled a real-world, all-comer patient population also showed the safety and efficacy of SCB, both in patients with ISR or *de novo* lesions (75, 76). The Nanolutè study evaluated the clinical performance of a novel SCB (Concept Medical Research Private Limited, India) for the treatment of ISR and *de novo* coronary lesions. Dual antiplatelet therapy was recommended for 3–12 months in this trial (75). The MACE rate was 4.2% with 3.2% TLR at 2 years. The EASTBOURNE registry also evaluated this kind of SCB and obtained similar results as the Nanolutè study (76). In this trial, DAPT duration was prescribed for a minimum of 1 and 6 months in the case of additional stent implantation. As for ACS patients, DAPT duration was prescribed according to the current guidelines.

## Conclusions and Perspective

**Figure 2:**
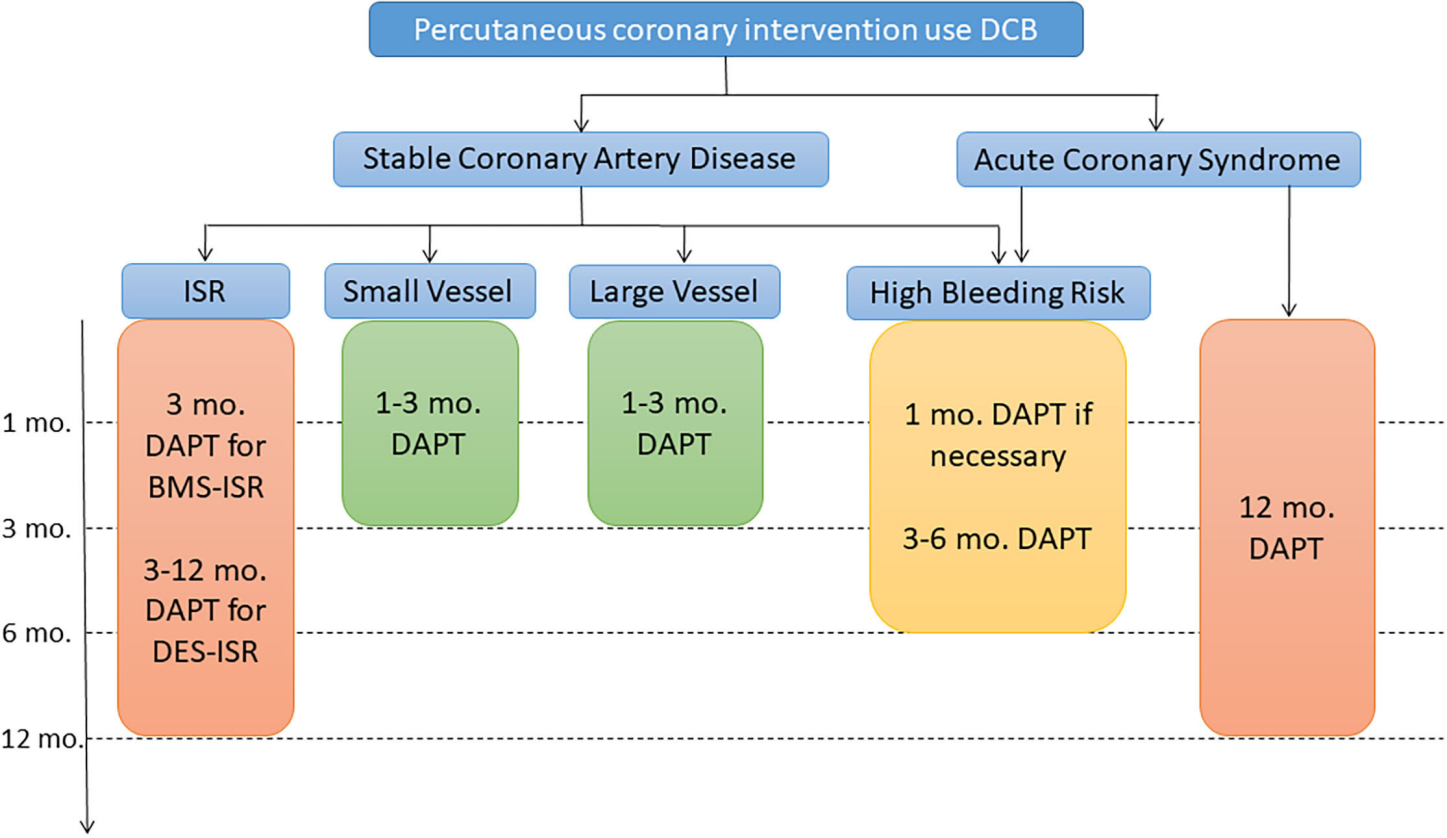
Algorithm for dual antiplatelet therapy (DAPT) in patients treated with percutaneous coronary intervention and only using DCB. DCB, drug-coated balloon; ISR, in-stent restenosis; CTO, chronic total occlusions. High bleeding risk is considered an increased risk of spontaneous bleeding during DAPT (e.g., PRECISE-DTPA score ≥25).

As DCB technology is playing an increasingly important role in PCI, standardizing postoperative drug treatment is essential. Defining the optimal duration of DAPT after DCB-only angioplasty remains an interesting question but the currently available evidence is limited. Here, we give a simple summary with suggestions for DAPT duration in the different clinical scenarios based on current evidence (Figure 2).

For a decade, we have been passionate about defining the optimal duration of DAPT after stenting and have conducted many RCTs. Now, it is time to focus on the optimal duration of DAPT after DCB-only angioplasty. Meanwhile, with the advancement of DCB technology and the discovery of potent antiplatelet drugs, the DAPT approach may shift to a new paradigm of single antiplatelet therapy. It is necessary to explore the feasibility of single antiplatelet therapy after DCB-only angioplasty.

## Author Contributions

All authors contributed to the manuscript production and in the final revision. YZ, QD, DC, and YX structured the manuscript giving contribute to table, figures, and text editing. JJ, XZ, and YZ revisited the article implementing the final manuscript form.

## Funding

JJ was supported by grant from the National Natural Science Foundation of China (No. 82170332) and Key Research and Development Program of Zhejiang Province (No. 2020C03016). XZ was supported by grant from the National Natural Science Foundation for young scientists of China (No. 82100346).

## Conflict of Interest

The authors declare that the research was conducted in the absence of any commercial or financial relationships that could be construed as a potential conflict of interest.

## Publisher's Note

All claims expressed in this article are solely those of the authors and do not necessarily represent those of their affiliated organizations, or those of the publisher, the editors and the reviewers. Any product that may be evaluated in this article, or claim that may be made by its manufacturer, is not guaranteed or endorsed by the publisher.
